# Surgical treatment of esophageal perforation after stereotactic body radiotherapy: A report of two cases

**DOI:** 10.1016/j.ijscr.2022.107805

**Published:** 2022-11-30

**Authors:** Takeharu Kato, Yoshihiro Kazama, Sho Matsuura, Sakae Nagaoka

**Affiliations:** Department of Gastroesophageal Surgery, Japanese Red Cross Medical Center, 4-1-22, Hiroo, Shibuya-Ku, Tokyo 150-8935, Japan

**Keywords:** Stereotactic body radiation therapy, CyberKnife, Esophageal perforation, Late esophageal toxicity, Vascular endothelial growth factor inhibitor, Esophagectomy

## Abstract

**Introduction and importance:**

Esophageal perforation due to stereotactic body radiotherapy (SBRT) is rare, and there is no consensus on the treatment strategy. Here, we report two cases of esophageal perforation caused by CyberKnife irradiation managed with distinct surgical approaches.

**Case presentation:**

Case 1 was a 54-year-old woman who was administered chemotherapy including bevacizumab and underwent CyberKnife SBRT for postoperative ovarian cancer (pStage IIIc) with metastasis in the eighth thoracic vertebra. Thirteen months after irradiation, she suddenly developed right back and anterior thoracic pain and was diagnosed with esophageal perforation. Despite open chest drainage and intercostal muscle (ICM) flap coverage, the fistula could not be closed, leading to pyogenic spondylitis and epidural abscess.

Case 2 was of a 58-year-old woman with mediastinal lymph node metastasis 5 years after uterine cancer surgery (pStage Ia) who underwent CyberKnife SBRT. Six months after irradiation, she experienced back pain and was diagnosed with esophageal perforation. After curative esophagectomy, the patient was discharged on postoperative day 22 without any adverse effects.

**Clinical discussion:**

Esophageal perforation by SBRT with vascular endothelial growth factor inhibitors (VEGFI) such as bevacizumab has rarely been reported. Considering the impaired wound healing system and blood perfusion caused by radiation therapy and VEGFI, difficulty closing the perforation covered with an ICM flap was hypothesized.

**Conclusion:**

Late esophageal toxicity from irradiation may cause impaired blood flow and wound healing; therefore, curative esophagectomy, including at the perforation site, is effective.

## Highlights

• We noted two cases of esophageal perforation caused by CyberKnife irradiation.
• A 54-year-old woman underwent CyberKnife SBRT for postoperative ovarian cancer.
• A 58-year-old woman underwent CyberKnife SBRT for mediastinal-lymph node metastasis.
• Irradiation may impair blood flow and wound healing.
• Curative esophagectomy, including at the perforation site, is effective.

## 1 Introduction

Stereotactic radiation therapy (SRT) is an irradiation method that precisely aligns radiation to the shape of the lesion and minimizes unnecessary radiation exposure when compared with conventional external beam radiation therapy (EBRT). Stereotactic body radiation therapy (SBRT), such as CyberKnife (Accuray Inc., Sunnyvale, CA, USA), is equipped with a lesion tracking system that evaluates body movements during irradiation and has been increasingly applied to irradiate tumors in the trunk of the body [1], [2].

SBRT has been reported to have adverse effects, causing ulceration, perforation, and bleeding, in cases of lesion proximity to the esophagus. Mild to severe esophagitis is common, and esophageal ulcers, perforation, and tracheoesophageal fistulas may occur in rare cases [3], [4], [5], [6].

We report two cases of esophageal perforation caused by CyberKnife irradiation. One is a case of esophageal perforation 13 months after CyberKnife irradiation conducted for the eighth thoracic metastasis of ovarian cancer, where the perforation site was covered with an intercostal muscle (ICM) flap. The other is a case of esophageal perforation 6 months after irradiation of mediastinal lymph node metastasis of uterine cancer, with subtotal esophagectomy.

The work has been reported in accordance with the SCARE 2020 criteria [7].

## 2 Presentation of cases

### 2.1 Case 1

A 54-year-old woman with post-surgery ovarian cancer (pStage IIIc) metastasized to the eighth thoracic vertebra was urgently brought to the hospital complaining of right back and anterior thoracic pain.

Two years prior, she had undergone a curative resection for ovarian cancer and six cycles of adjuvant chemotherapy. After eight months, positron emission tomography-computed tomography (PET-CT) showed a pelvic recurrence, and she received three courses of chemotherapy, including bevacizumab. Four months later, metastasis to the eighth thoracic vertebra appeared, and she received CyberKnife 30 Gy/3 fractions (fr) irradiation at the same site and continued the bevacizumab containing regimen. Since esophagitis or other symptoms were not suspected, a close examination of the upper gastrointestinal tract before and during CyberKnife was not performed.

**Fig. 1:**
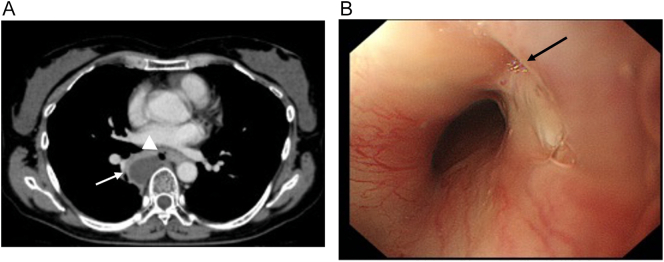
Preoperative findings. A) Contrast-enhanced computed tomography scan showed a 30 × 25 mm encapsulated fluid retention in the mediastinum (closed arrow) and a bubble near the esophagus (crossed arrowhead). B) Upper gastrointestinal endoscopy revealed a fistula at the 2 o'clock position, 30 cm from the incisor (closed arrow).

On arrival at our hospital, the patient had a fever of 39 °C and her C-reactive protein (CRP) level was 17.8 mg/dL. A contrast-enhanced CT scan revealed a mediastinal abscess (Fig. 1A), and intravenous antibiotic therapy was initiated. Considering that her blood test findings did not improve, on the fourth day of hospitalization we performed upper gastrointestinal endoscopy, which revealed perforation of the esophagus (Fig. 1B), and emergency surgery was planned.

#### 2.1.1 Surgical findings

**Fig. 2:**
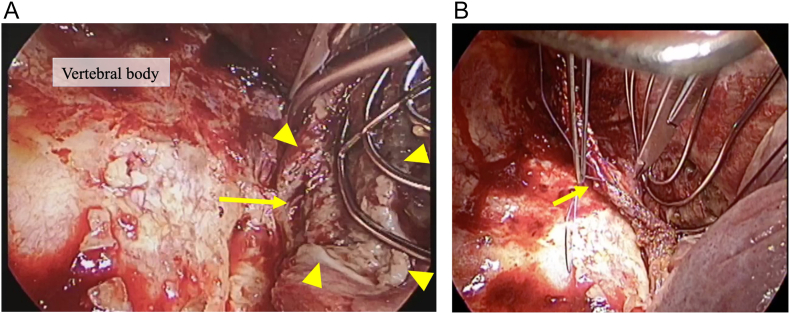
Intraoperative findings. A) The perforation site (closed arrow) and necrotic esophageal adventitia and muscularis propria (closed arrowhead) were detected. B) The perforation site was covered with an intercostal muscle flap (closed arrow).

Chest drainage via thoracotomy and a pedicled ICM flap was performed. The patient was placed in the left lateral recumbent position and the chest wall was opened. An encapsulated abscess with whitish-yellow fluid was identified when the pleura was dissected towards the dorsal side and separated from the vertebral body. The esophageal adventitia and muscularis propria of the perforation site showed lytic necrosis over one vertebral body (Fig. 2A). Simple closure was not feasible and the perforation site was covered with an ICM flap (Fig. 2B).

#### 2.1.2 Postoperative course

On postoperative day (POD) 7, CT-guided drainage was performed for an abscess around the perforation site. The patient was discharged with a drain on POD 71. Three months after surgery, infection from the abscess cavity around the perforation site spread to the spine and the patient developed pyogenic spondylitis. Six months after surgery, spinal epidural abscess was diagnosed by posterior decompression at Th8–9. Simultaneously, an esophageal bypass was performed for the tracheoesophageal fistula. The patient died of disease aggravation ten months after the initial surgery.

### 2.2 Case 2

**Fig. 3:**
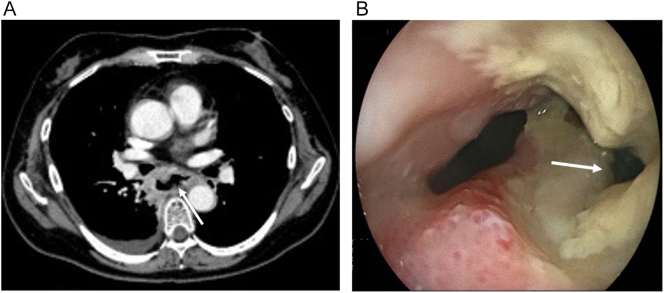
Preoperative findings. A) The mid-thoracic esophagus was torn, the continuous gas image from the perforation site was encapsulated, and mediastinal emphysema was localized (closed arrowhead). B) Upper gastrointestinal endoscopy revealed a large esophageal ulceration, 0–6 o'clock, 30–33 cm from the incisor tooth. The ulcer was perforated at the 4 o'clock position, and its tip was suspected to have reached the right mediastinal pleura (closed arrow).

A 58-year-old woman with post-surgery uterine cancer (pStage Ia) was diagnosed with mediastinal lymph node metastasis and received CyberKnife 35 Gy/5 fr irradiation. There were no symptomatic esophageal signs, and hence no esophageal workup was performed before and during radiotherapy. Six months after irradiation, she was admitted to hospital with complaints of chest and back pain and cough. The blood test showed a white blood cell count of 11,860/μL and a CRP level of 11.4 mg/dL. CT showed pleural effusion in the right lung, disruption of the mid-thoracic esophagus, and localized mediastinal emphysema (Fig. 3A). Upper gastrointestinal endoscopy revealed a sizeable esophageal ulcer 30–33 cm from the incisor (Fig. 3B). The ulcer was perforated at the 4 o'clock position and it reached the right mediastinal pleura. Esophagectomy and gastric tube reconstruction were performed via the retrosternal route.

#### 2.2.1 Surgical findings

**Fig. 4:**
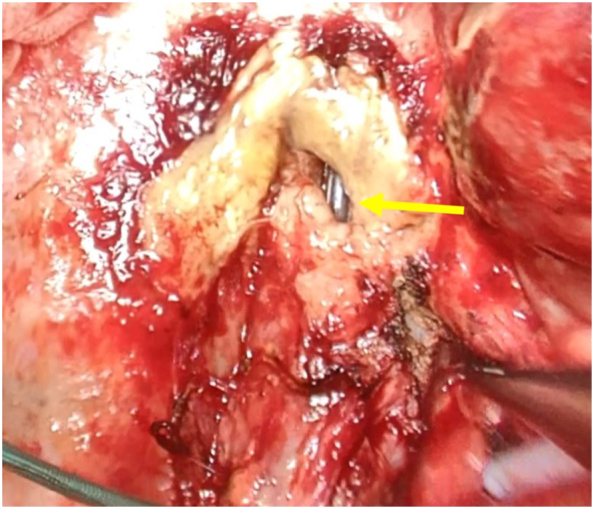
Intraoperative findings. Esophageal perforation is identified in the middle mediastinum with yellow fibrin clots (closed arrow).

The patient was positioned in the lateral decubitus position, and posterolateral thoracotomy was performed. Esophageal perforation (1.5 cm) was identified in the middle mediastinum, with yellow fibrin clots adhering to the surrounding tissue (Fig. 4). Esophagectomy was performed, followed by reconstruction using a gastric tube through the retrosternal route.

#### 2.2.2 Histological findings

**Fig. 5:**
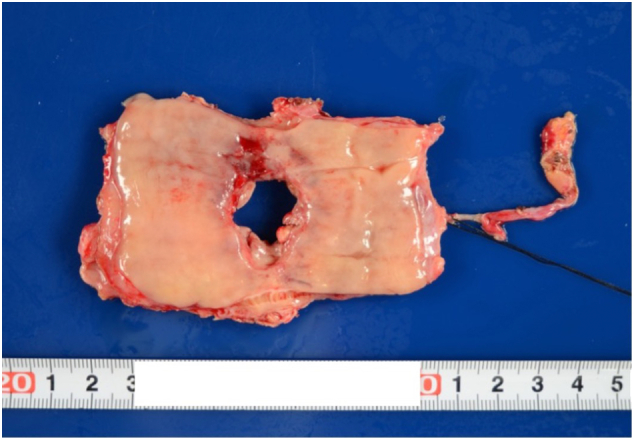
Histological findings. Extracted specimen: A perforation with a maximum diameter of 1.7 cm was identified in the right lateral wall of the esophagus.

A 1.7 cm diameter perforation site was observed on the right wall of the esophagus (Fig. 5).

#### 2.2.3 Postoperative course

No adverse effects were observed and on POD 7, gastrografin swallow showed no anastomotic leakage. The patient resumed oral intake and was discharged on POD 22.

## 3 Discussion

Esophageal perforation can occur for a variety of reasons, including an obstructed esophagus due to pre-existing esophageal diseases, iatrogenic causes, trauma, swallowing of foreign bodies, and spontaneous rupture [8]. While the mortality rate is 10 %–40 % [9], there is no standardized treatment and, moreover, customized treatments and strategies are needed. Careful decision-making is required to determine whether conservative or surgical treatment is appropriate. Considering that esophageal surgery is highly invasive, complications can result with seriously detrimental outcomes. The frequency of radiation-related esophageal perforation as a complication of radical chemoradiotherapy for esophageal cancer has been reported in 0.4 %–1 % of cases [10]. There have been reports of perforation as a late complication several years after radical or adjuvant chemoradiotherapy, as well as when the radiation dose reaches 14–58 Gy. Because of fibrosis and radiation-induced impaired healing, achieving a complete cure with conservative treatment is difficult.

Relative 3D-Conformal Radiation Therapy (CRT), using the CyberKnife, was reported to reduce toxicity to surrounding organs [11]. Late esophageal toxicity (LET) is defined as that commonly occurring 3–18 months after radiation therapy, and SBRT irradiation for a maximum point dose over 27 Gy/3 fr or 35 Gy/5 fr to normal esophageal tissue is associated with the thresholds for risk of stenosis or perforation as an LET [12], [13]. It has also been suggested that concurrent or iatrogenic use of vascular endothelial growth factor inhibitors (VEGFI) such as bevacizumab, may be a risk factor for gastrointestinal (GI) perforation in patients undergoing SBRT [14]. VEGFI therapy has been associated with a 1 %–2 % risk of GI perforation, whereas the crude rate of GI perforation with VEGFI therapy after SBRT was 15 %. This was hypothesized to be related to inadequate repair of radiation damage due to impaired VEGF response [14], [15].

In Case 1, a high dose of CyberKnife 30 Gy/3 fr to the esophagus with VEGFI therapy may have caused esophageal perforation. The fistula could not be closed by covering with an ICM flap because of impaired wound healing associated with irradiation. Pyogenic spondylitis and epidural abscess occurred because of uncontrolled infection, decreasing the quality of life.

In Case 2, the high irradiation dose of CyberKnife 35 Gy/5 fr in the proximity of the esophagus may have been the cause of esophageal perforation. Although a large perforation was detected by endoscopy, mediastinal emphysema was localized on the CT image, and the patient's general condition was preserved; therefore, a one-stage operation was performed based on the experience of Case 1. Postoperative infection control was good, and chemotherapy was administered for the underlying disease progression 6 months after discharge.

## 4 Conclusion

In this study, we report two cases of delayed esophageal perforation due to SBRT. This study indicates that curative esophagectomy may be effective for esophageal perforation as the LET after SBRT because of delayed wound healing and fibrosis of the peripheral tissue. Particularly when VEGFI therapy is combined with SBRT, as VEGFI may inhibit angiogenesis and cause ischemia of the esophagus, aggressive esophagectomy may be a viable option if the patient's ability to tolerate the operation is acceptable.

## Provenance and peer review

Not commissioned, externally peer-reviewed.

## Consent

Written informed consent was obtained from the patient for publication of this case report and accompanying images. A copy of the written consent is available for review by the Editor-in-Chief of this journal on request.

## Ethical approval

N/A.

## Funding

This research did not receive any specific grant from funding agencies in the public, commercial, or not-for-profit sectors.

## Guarantor

Takeharu Kato.

## Research registration number

N/A.

## CRediT authorship contribution statement

Takeharu Kato – Conceptualization and drafting of the case report.
Yoshihiro Kazama – Review of literature.
Sho Matsuura – Compilation of data.
Sakae Nagaoka – Surgery and its procedure.

## Conflict of interest

The authors report no declarations of interest.
